# Posterior muscle chain activity during various extension exercises: an observational study

**DOI:** 10.1186/1471-2474-14-204

**Published:** 2013-07-09

**Authors:** Eline MD De Ridder, Jessica O Van Oosterwijck, Andry Vleeming, Guy G Vanderstraeten, Lieven A Danneels

**Affiliations:** 1 Department of Rehabilitation Sciences and Physical Therapy, Faculty of Medicine and Health Sciences, Ghent University, Ghent, Belgium, UK; 2 Department of Anatomy, Faculty of Medicine and Allied Health Sciences, Erasmus University Rotterdam, Rotterdam, The Netherlands

**Keywords:** Trunk extensor muscles, Multifidus, Posterior muscle chain, Extension exercise, Electromyography, Spine

## Abstract

**Background:**

Back extension exercises are often used in the rehabilitation of low back pain. However, at present it is not clear how the posterior muscles are recruited during different types of extension exercises. Therefore, the present study will evaluate the myoelectric activity of thoracic, lumbar and hip extensor muscles during different extension exercises in healthy persons. Based on these physiological observations we will make recommendations regarding the use of extensions exercises in clinical practice.

**Methods:**

Fourteen healthy subjects performed four standardized extension exercises (dynamic trunk extension, dynamic-static trunk extension, dynamic leg extension, dynamic-static leg extension) in randomized order at an intensity of 60% of 1-RM (one repetition maximum). Surface EMG signals of Latissimus dorsi (LD), Longissimus thoracis pars thoracic (LTT) and lumborum (LTL), Iliocostalis lumborum pars thoracic (ILT) and lumborum (ILL), lumbar Multifidus (LM) and Gluteus Maximus (GM) were measured during the various exercises. Subsequently, EMG root mean square values were calculated and compared between trunk and leg extension exercises, as well as between a dynamic and dynamic-static performance using mixed model analysis. During the dynamic exercises a 2 second concentric contraction was followed by a 2 second eccentric contraction, whereas in the dynamic-static performance, a 5 second isometric interval was added in between the concentric and eccentric contraction phase.

**Results:**

In general, the muscles of the posterior chain were recruited on a higher level during trunk extension (mean ± SD, 56.6 ± 30.8%MVC) compared to leg extension (47.4 ± 30.3%MVC) (p ≤ 0.001). No significant differences were found in mean muscle activity between dynamic and dynamic-static performances (p = 0.053). The thoracic muscles (LTT and ILT) were recruited more during trunk extension (64.9 ± 27.1%MVC) than during leg extension (54.2 ± 22.1%MVC) (p = 0.045) without significant differences in activity between both muscles (p = 0.138). There was no significant differences in thoracic muscle usage between the dynamic or dynamic-static performance of the extension exercises (p = 0.574).

Lumbar muscle activity (LTT, ILL, LM) was higher during trunk extension (70.6 ± 22.2%MVC) compared to leg extension (61.7 ± 27.0%MVC) (p = 0.047). No differences in myoelectric activity between the lumbar muscles could be demonstrated during the extension exercises (p = 0.574). During each exercise the LD (19.2 ± 13.9%MVC) and GM (28.2 ± 14.6%MVC) were recruited significantly less than the thoracic and lumbar muscles.

**Conclusion:**

The recruitment of the posterior muscle chain during different types of extension exercises was influenced by the moving body part, but not by the type of contraction. All muscle groups were activated at a higher degree during trunk extension compared to leg extension. Based on the recruitment level of the different muscles, all exercises can be used to improve the endurance capacity of thoracic muscles, however for improvement of lumbar muscle endurance leg extension exercises seem to be more appropriate. To train the endurance capacity of the LD and GM extension exercises are not appropriate.

## Background

The posterior spine muscle chain consists of the thoracic, lumbar and hip extensor muscles. Optimal condition of this muscle chain includes optimal motor control, strength and endurance, and is a prerequisite in the prevention and treatment of low back pain (LBP) in non-athlete and athlete populations [1-3]. Many studies report motor control impairment, decreased muscle strength and endurance in LBP patients [4-12]. With regard to muscle endurance, researchers have found lower endurance times in LBP patients compared to healthy persons [13]. Furthermore Biering-Sorensen reports, that isometric back muscle endurance is a significant predictor of first-time occurrence of LBP among men, and of recurrent LBP [7]. The produced strength of the trunk extensors seems to be less useful for discriminating between healthy people and LBP patients than endurance capacity. Nonetheless, Luoto et al. [6] report that those with poor back muscle strength were 3 times more likely to develop LBP than those with good back muscle strength. Among athletes, sport induced muscles imbalances within the trunk muscles or hip muscles, seems to be related to LBP, due to abnormal spinal loading [14,15]. This implies that a good condition of the posterior muscle chain and a good balance between the lumbar, thoracic and hip extensors is crucial.

Literature provides evidence that endurance and strength training of the trunk extensors is important in the prevention and treatment of LBP [16,17]. Exercise will lead to a decrease in pain and disability, and to a reduction of LBP occurrence among athletes [3,14,15]. Moreover Durall et al. [3] demonstrated that pre-season strength training of the trunk extensors is also beneficial for sport performance in gymnasts.

Although several resistance training exercises have been proposed to improve strength and/or endurance of the back muscles, there is little agreement upon which exercises are the most effective [9,17-20]. Extension exercises performed in prone position are frequently described in the literature [18,21-26]. For example prone arch exercises, i.e. combined trunk and leg extension, activate the back muscles at a high level. However, this type of exercise will also cause high spinal compressive loads due to hyperlordosis of the spine [18]. Therefore exercises in which only the subject’s trunk or legs are unsupported, and the neutral lordosis of the low back is sustained, are assumed to be safer [18,27]. This type of exercise will activate the back muscles at 40–70% of their maximal voluntary contraction (MVC) [26,28,29].

Several studies describe that in addition to the thoracic and lumbar muscles, the Latissimus dorsi (LD) and hip extensors contribute during trunk extension performance [28,30-33]. These findings emphasize the importance of a global view on the contribution of various, relevant muscles, when evaluating muscle activity during exercise.

To our knowledge only Plamondon et al. [28] have investigated if differences exist in lumbar muscle activity and the hip extensors during trunk and leg extension exercises in a healthy population. The authors reported that no differences were observed between the two different types of extension exercise regarding activity of the erector spinae (ES), the multifidus (LM), and the gluteus maximus (GM). This study however did not evaluate the thoracic muscles.

With regard to contraction modalities, back extension exercises can be performed in a static [7,13,21,32-38], dynamic [21,23,28,30,31,38-43], or dynamic-static way [23,28,30,44]. Plamondon et al. [28] described that during the dynamic phase of a trunk extension exercise, the lumbar ES were activated to a higher degree than during the static phase. Furthermore the LM seemed to be less active during isometric trunk extension than during dynamic trunk extension [21]. From the perspective of rehabilitation, a recent study has demonstrated that performing dynamic–static exercises during LBP rehabilitation will result in a better long term outcome compared to dynamic exercises [22,45]. Although, the type of contraction seems to play a role in muscle training, at present there are no studies available which have investigated the influence of the contraction modality on the recruitment of the posterior muscle chain.

In order to create specific exercise programs for both elite sportsmen and LBP patients, insights into the relative contribution of the different muscles of the posterior spine muscle chain in healthy persons, during different extension and contraction modalities, are required. This study will be the first to evaluate the recruitment of the hip, lumbar and thoracic trunk muscles during various extension exercises in healthy subjects. Therefore the global posterior spine muscle chain will be evaluated during trunk and leg extensions, and during different contraction modalities (i.e. dynamic and dynamic-static).

## Methods

### Subjects

Fourteen healthy subjects (6 females, 8 males), with a mean age of 24.7 years and a standard deviation of ±3.2 years volunteered for this study. Subjects had a mean height of 172.9 ±6.4 cm, and mean weight of 64.5 ±12.5 kg, mean Body Mass Index (BMI) of 23,0 ±3.1 kg/m^2^. Subjects were recruited by an advertisement which was spread amongst students and employees from Ghent University and Ghent university Hospital. Exclusion criteria for study participation were a medical consultation for LBP in the past year, current back pain, previous back surgery and spinal deformities. All subjects received a leaflet containing information about the study procedure and were asked to sign the informed consent upon agreement of study participation. The study protocol, information leaflet and informed consent were approved by the local Ethics Committee (Ghent university hospital).

### General design

Each subject attended a first testing session, to determine the 1 repetition maximum (1-RM) for each exercise. This was followed by two exercise sessions in which standardized trunk extensions were performed at 60% of 1-RM. The sequence of the 4 exercises was randomized using lottery, and then distributed among the 2 sessions (2 in each session), with at least two days in between the different sessions.

Surface electromyography (sEMG) of the hip, lumbar and thoracic trunk muscles was used to evaluate the muscle activity of the global posterior chain during different modalities of extension exercises. Differentiation between the lumbar and thoracic back muscles was made by detailed electrode placement based on previous work [22]. Each exercise session consisted of the electrode placement, measuring the MVC of the different muscles, and the performance of the two different extension exercise modalities.

### Electromyography

The sEMG signals of 7 muscles, were bilaterally measured using a 16 channel telemetric surface EMG system (TeleMyo 2400 G2 Telemetry System, Noraxon, USA). To reduce skin impedance and to improve skin contact, the skin was prepared by shaving and rubbing the skin with alcohol. After skin preparation, 7 pairs of surface electrodes (Noraxon dual electrodes) were bilaterally attached, parallel to the muscle fiber orientation over the following muscles [32,46,47]; Gluteus maximus (GM) (midway between the posterosuperior iliac spine and the ischial tuberosity), lumbar Multifidus (LM) (2 cm lateral to the midline of the body, above and below a line connecting both posterior superior iliac spines), Latissimus dorsi (LD) (3 cm lateral and caudal to the angulus inferior of the scapula), Longissimus thoracis pars thoracic (LTT) (at the L1 level, midway between the line through the spinous process and a vertical line through the posterior superior iliac spine), Longissimus thoracis pars lumborum (LTL) (lateral at the intersection of a horizontal line through the spinous process of L5 and a line between the interspinous space of L1–L2 and the posterosuperior iliac spine), Iliocostalis lumborum pars thoracis (ILT) (at the L1 level, midway between the lateral palpable border of the erector spinae and a vertical line through the posterosuperior iliac spine), and Iliocostalis lumborum pars lumborum (ILL) (at the L4 level, midway between the lateral palpable border of the erector spinae and a vertical line through the posterosuperior iliac spine).

A reference electrode was placed on the angulus inferior of the scapula. The electrodes had a fixed inter-electrode distance of 2 cm and an electric surface contact of 1 cm diameter.

The raw signals were bandpass-filtered between 10 and 500 Hz, amplified (common mode rejection ratio >100 dB, overall gain 1000, noise <1 uV Root mean square (RMS)), and analogue-to-digital (16-bit) converted at a sampling rate of 1500 Hz. The signal processing consisted of full wave rectification and smoothing, using a root mean square algorithm with a 100 ms time constant. The RMS is a real time indicator of the amount of electric activity of the investigated muscle.

Muscle activity was measured during all contraction phases of the exercise. As the first repetition was considered as a familiarization repetition, the mean muscle activity level (across all contraction phases) was measured over repetition 2–6 (5 repetitions) and used for further analysis.

### Determination of the exercise intensity (60%RM)

The exercise intensity for this protocol was set at 60% of 1-RM. The Repetition Maximum represents the maximum number of repetitions performed before fatigue prohibits completion of an additional repetition and generally reflects the intensity of the exercise [45]. The 1-RM was determined for every patient and each exercise during the testing session which took place minimum three days before the first exercise session. To determine the exercise load, all subjects performed a maximal test in which they were asked to execute the maximal amount of repetitions of the dynamic trunk/leg extension with the weight of their upper/lower body as the exercise weight (which is estimated as 70% and 30% of the total body weight respectively). The number of repetitions each subject was able to perform during both types of exercises, using this method, was registered.

The exercise intensity was individually calculated using the following formula [47]:

$$\begin{array}{rl} \left(\mathrm{Upper}\right) & \left(/\mathrm{lower}\;\mathrm{body}\;\mathrm{weight}\;\left[\mathrm{kg}\right]\times \mathrm{Exercise}\;\mathrm{load}\;\left[60\%\mathrm{RM}\right]\right)\\ & /\mathrm{Exercise}\;\mathrm{load}\;\mathrm{determined}\;\mathrm{on}\;\mathrm{testing}\;\mathrm{day}\\ & \left[\mathrm{Holten}-\mathrm{diagram}\right] \end{array}$$

Weight adjustments or assistance during exercises are displayed in Table 1.

**Table 1:** Adjusted ( +) or assistance (−) weight during the different exercises per subject

	**Dyn Trunk**	**Dyn-stat trunk**	**Dyn leg**	**Dyn-stat leg**
1	+0	−1,5	+6	−1
2	+4	−1	+6.5	−0.5
3	−1	−17	−0.5	−3
4	+14	−2	+5.5	+1
5	+1	−5	+5.5	+3
6	+1	−5	+3	−1.5
7	+8.5	+5	+7	−0.5
8	+10	+4	+11	+3.5
9	−6	−7	−1	−4
10	+22.5	−2.5	+10.5	+0.5
11	+4	+0.0	+4.5	+0,0
12	−1.5	−6.5	+9	+5.5
13	+9	+1	+6	−2
14	+5	−3	+5.5	+0.5
**MEAN**	**+5**	**−3**	**+5.5**	**0**

Accurate to 0,5 kg.

*Dyn trunk* (dynamic trunk extension), *Dyn-stat trunk* (dynamic-static trunk extension), *Dyn leg* (dynamic leg extension), *Dyn-stat leg* (Dynamic-static leg extension).

### Exercise protocol

#### Maximal voluntary contraction (MVC)

In order to compare the muscle activity between muscles, the sEMG data were normalized against their MVC. Before starting the exercises, the MVC’s for the back and hip muscles were measured 3 times during 4 seconds, with 30 seconds of rest between each trial. All tests were performed in prone position. Since the Intraclass Correlation Coefficients (ICC (2,1)) of the MVC’s were found to be high (0,78 - 0,91), the average MVC from each muscle was used for further analysis.

To obtain the MVC of the GM, the knee of the tested side was flexed 90°. The opposite leg was strapped to the table. Maximal resistance against hip extension was given proximal of the knee joint. To measure the MVC of the LD, subjects were lying with their arms in endorotation. Maximal resistance was given proximal of the elbows against retroflexion of the arm [48]. To measure the MVC of the trunk extensors, subjects lay in prone position and had to place the back of their hands on their forehead. The legs were strapped to the table at the middle of the calfs. Maximal resistance was given against trunk extension on the angulus inferior of both scapulae [48,49].

#### Extension exercises

The exercise protocol consisted of four exercises including dynamic trunk extension, dynamic-static trunk extension, dynamic leg extension and dynamic-static leg extension. Between the exercises, a resting period of 40 minutes in lying position, was obligated to prevent muscular fatigue.

**Figure 1:**
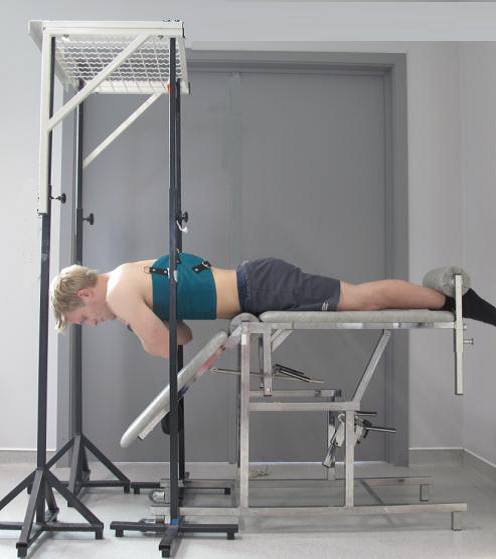
Position trunk extension exercises.

In order to perform the trunk extension, subjects were placed in prone position, with the upper body free from the couch, and the superior border of the anterior iliac on the edge of the couch [47]. Their legs were strapped to the couch at the ankles, and hands were placed crossed on the shoulders. The subjects were instructed to raise their upper body from the starting position, i.e. 45° flexion, to horizontal, while looking downward at a visual fixation point. The trunk extension exercise is represented in Figure 1.

**Figure 2:**
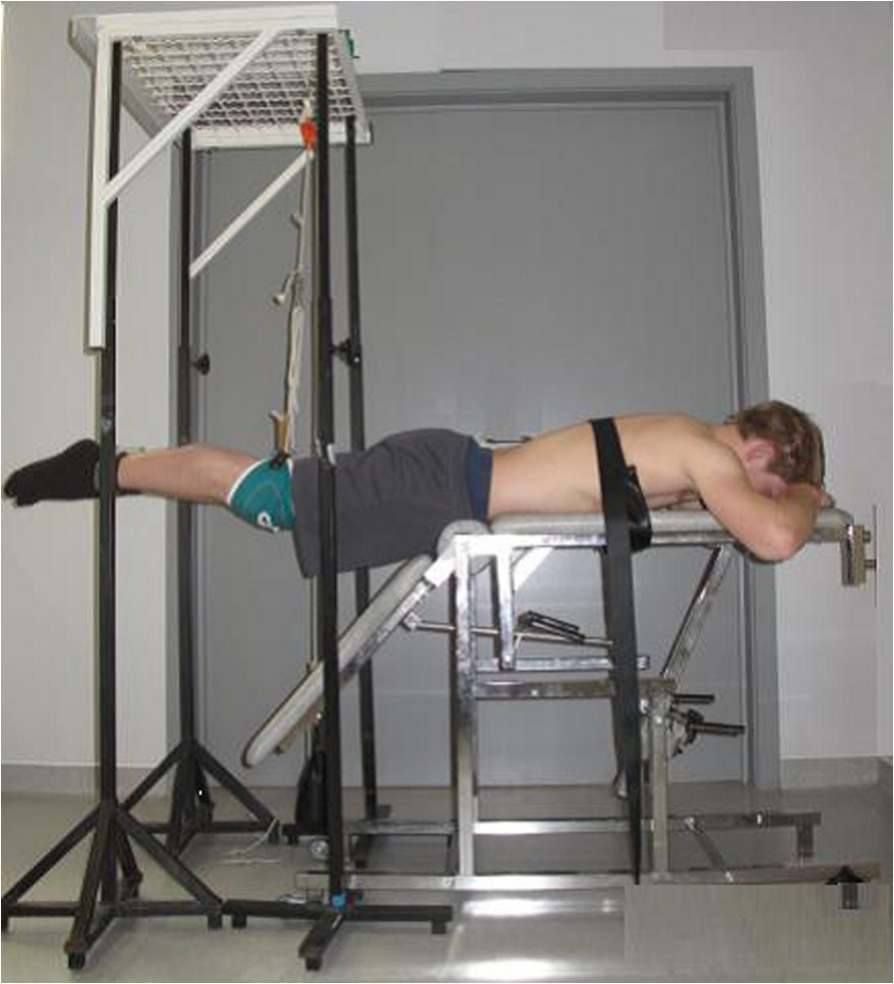
Position leg extension exercises.

The leg extension exercise was also performed in prone position on a couch (Figure 2). The upper body was strapped to the couch with a belt at the level of the angulus inferior of the scapulae, and hands were positioned under the forehead. The subjects were instructed to lift both legs from the starting position of 45°flexion, to the horizontal.

Both exercises were performed in a dynamic and dynamic-static manner. During the dynamic modality, one repetition consisted of 2 seconds in which the upper body or legs were raised, and 2 seconds during which the upper body or legs were lowered to the start position [42]. During the dynamic-static exercise, the upper body or legs were held in horizontal position during 5 seconds, between the concentric and eccentric phase.

During all exercises, tactile feedback was given by a rope between the two vertical stands to which indicated that the horizontal position had been reached. A metronome (60 beats/min) was used to ensure appropriate timing for the contractions. After each exercise patients assessed the intensity of the exercise by verbally providing a Borg score. The Borg scale measures perceived exertion on a scale from 6–20 (6 = no exertion at all, 20 = maximal exertion).

### Statistical analysis

A mixed model analysis, was conducted with SPSS 16 for Windows (SPSS Inc. Headquarters, Chicago, Illinois), to investigate the influence of 4 independent factors on the posterior chain muscle activity. Following factors were used: factor muscle (7 different muscles), factor side (left and right muscle activity), factor body part (trunk vs leg extension), factor contraction type (dynamic vs static-dynamic extension).

Post hoc comparisons were made with Bonferonni corrections. Because post hoc analysis showed differences between muscles, a second mixed model was performed with the thoracic muscles apart and a third with the lumbar muscles separately. An additional mixed model analysis with factors body part, contraction type and contraction phase (concentric, isometric and eccentric) was conducted for each muscle separately, to investigate the differences in mean muscle activity during the different phases of contraction. Statistical significance for all tests was accepted at the 5% level.

## Results

A mixed model analysis showed no significant differences between left and right muscle activity for each exercise, therefore mean muscle activity of both sides for each muscle and exercise was calculated and described in Table 2. Mean muscle activity never exceeded 78% of the MVC.

**Table 2:** Mean muscle activity (± standard deviation) for each muscle during the four extension exercises (%MVC)

		**LD**	**LTT**	**ILT**	**LTL**	**ILL**	**LM**	**GM**
	**Dyn trunk**	27.38 ± 6.94	79.03 ± 31.51	64.12 ± 20.72	89.07 ± 19.49	91.47 ± 25.60	78.69 ± 23.88	44.87 ± 30.03
**Concentric phase**	**Dyn-stat trunk**	19.43 ± 2.73	69.64 ± 14.57	77.87 ± 23.19	86.90 ± 29.9	75.04 ± 28.92	82.00 ± 30.57	38.18 ± 23.18
	**Dyn leg**	9.72 ± 3.44	67.84 ± 20.68	62.95 ± 10.26	75.24 ± 20.82	88.25 ± 21.64	55.39 ± 8.57	30.33 ± 16.18
	**Dyn-stat leg**	22.64 ± 11.01	81.93 ± 26.91	63.16 ± 31.14	81.36 ± 17.54	79.60 ± 22.21	65.10 ± 36.65	43.25 ± 19.38
	**Dyn trunk**	N.A.	N.A.	N.A.	N.A.	N.A.	N.A.	N.A.
**Isometric phase**	**Dyn-stat trunk**	19.30 ± 2.10	69.92 ± 26.48	74.05 ± 21.64	81.54 ± 25.49	70.12 ± 27.10	70.23 ± 29.52	35.49 ± 12.99
	**Dyn leg**	N.A.	N.A.	N.A.	N.A.	N.A.	N.A.	N.A.
	**Dyn-stat leg**	20.56 ± 11.63	66.23 ± 31.16	61.15 ± 27.07	77.00 ± 19.56	72.73 ± 21.67	65.77 ± 30.66	35.27 ± 30.29
	**Dyn trunk**	26.57 ± 7.09	52.67 ± 22.15	50.19 ± 15.19	59.45 ± 17.29	57.94 ± 15.53	62.35 ± 24.44	33.09 ± 15.25
**Eccentric phase**	**Dyn-stat trunk**	19.06 ± 3.97	60.17 ± 27.79	44.02 ± 15.61	57.56 ± 23.71	66.57 ± 23.00	56.15 ± 30.65	29.38 ± 19.62
	**Dyn leg**	9.84 ± 3.51	46.84 ± 14.60	44.22 ± 10.42	49.81 ± 19.81	53.20 ± 17.70	53.21 ± 34.36	20.33 ± 9.56
	**Dyn-stat leg**	20.85 ± 7.51	54.76 ± 10.05	51.16 ± 26.47	48.03 ± 11.03	47.71 ± 15.60	58.59 ± 30.25	14.94 ± 9.59

*LD* lattisimus dorsi, *LTT* longissimus thoracis pars thoracic, *LTL* longissimus thoracis pars lumborum, *ILT* Ilioctalis lumborum pars thoracis, *ILL* Iliocostalis Lumborum pars Lumborum, *LM* Lumbar Multifidus, *GM* Gluteus maximus.

*N.A* Data Not available.

### Recruitment of the posterior muscle chain

The model with averaged level of activity among sides showed no significant interaction between the main factors. The factor ‘muscle’ (p ≤ 0.001) and the factor ‘body part’(p ≤ 0.001) were significant, while the factor ‘contraction type’ (p = 0.053) was not.

**Figure 3:**
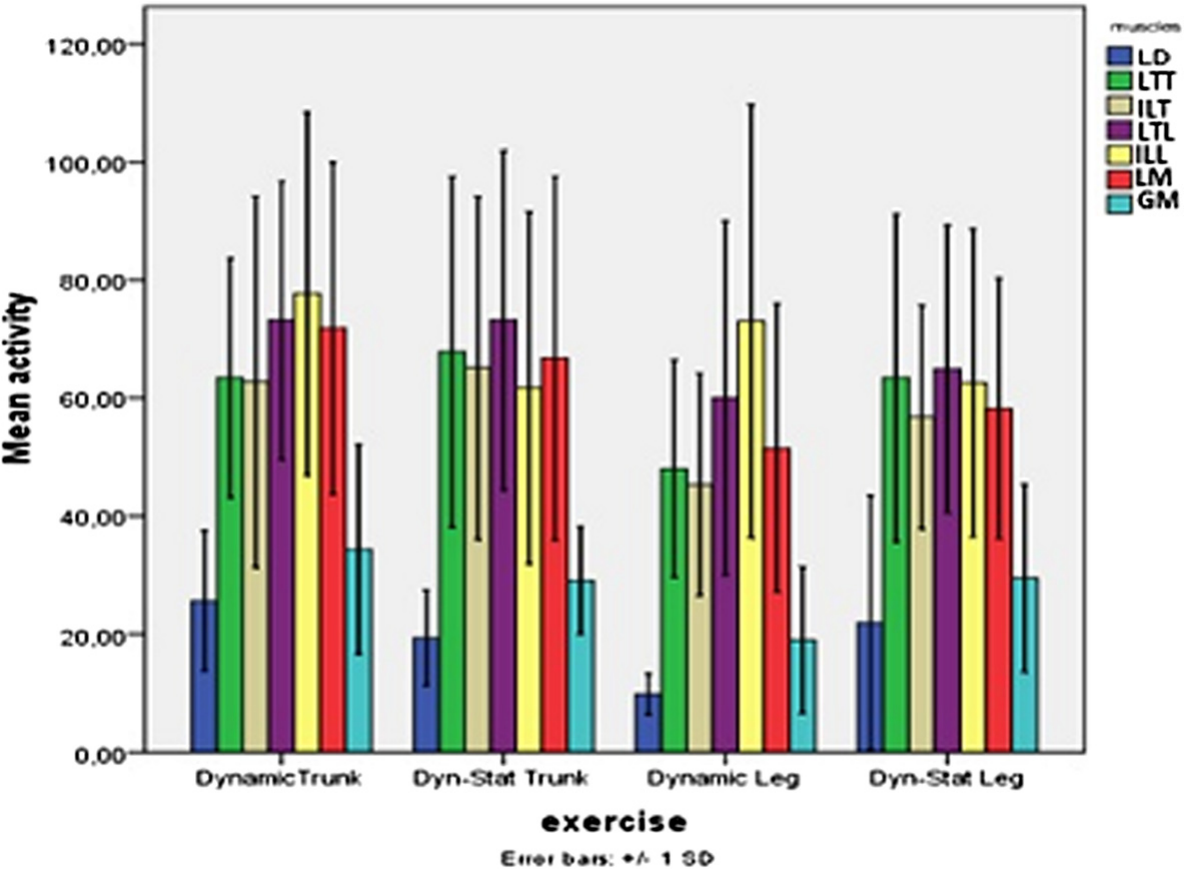
**Mean muscle activity (%MVC) and standard deviation for each muscle during four exercises.** LD = lattisimus dorsi, LTT = longissimus thoracis pars thoracic, LTL = longissimus thoracis pars lumborum, ILT = Ilioctalis lumborum pars thoracis, ILL = Iliocostalis lumborum pars lumborum, LM = Lumbar Multifidus, GM = Gluteus maximus.

Post hoc analysis for ‘muscle’ showed that both the LD and GM were recruited significantly less than the thoracic and lumbar muscles during each exercise. Further analysis of these muscles showed that the mean activity of the LD over all exercises was 19.4 ± 13.9%MVC, while the activity of the GM was slightly higher, namely 28.4 ± 14.6%MVC (p = 0.004) (Figure 3). The type of contraction or the moving body part had no significant influence on the activity of these muscles separately.

Post hoc analyses for ‘body part’ showed that the mean posterior spine muscle usage, was significantly higher during trunk extension (56.6 ± 30.8%MVC) than during leg extension exercises (47.4 ± 30.3%MVC) (Figure 3). Thus, independently of the investigated muscle, all muscles were recruited on a higher degree during trunk extension exercises.

For all muscles, except for the ILL, the lowest activity was found during dynamic leg extension (9.9-60.0%MVC), however no difference with dynamic – static leg extension (21.9-64.9%MVC) could be established.

Since the post hoc analysis showed that the LD and GM were recruited less than the paraspinal muscles, and given the anatomical and functional differences between the thoracic and lumbar muscles, two more mixed models were conducted without the LD and GM. One model included the thoracic muscles (LTT and ILT), while the other included the lumbar muscles (LM, LTL and ILL).

### Recruitment of the thoracic muscles of the posterior muscle chain

For the thoracic muscles, data showed that there was no significant interaction between the main factors. The factor ‘body part’ had a significant influence on the thoracic muscle activity (p = 0.045), while no significant effects could be established for the factors ‘muscle’ (p = 0.574) and ‘contraction type’ (p = 0.138).

This implicates that regarding the performance of the extension exercises, no differences in LTT and ILT activity could be established (Figure 3). Post hoc analysis for ‘body part’ revealed that the thoracic muscle activity was significant higher during trunk compared to leg extension (mean ± SD, 64.9 ± 27.1%MVC vs 54.2 ± 22.1%MVC).

### Recruitment of the lumbar muscles of the posterior muscle chain

When the lumbar muscles were examined separate no significant interaction effects were found between the main factors (p > 0.05), nonetheless the main effect ‘body part’ had a significant effect (p = 0.047) on the lumbar muscle activity. Lumbar muscle usage was higher during trunk extension (70.6 ± 22.2%MVC) compared to leg extension (61.7 ± 27.0%MVC).

No differences between the LM, ILL and LTL could be demonstrated during the extension exercises (p = 0.574). The mean activity level of the LM (62.1%MVC) was slightly, but not significant lower than the activity of the ILL (68.8%MVC) or LTL (67.8%MVC).

Furthermore performing the exercises in a dynamic or dynamic-static way did not have an influence on lumbar muscle activity (respectively 68.5%MVC vs 64.8%MVC).

### Recruitment of the posterior muscle chain: concentric, isometric and eccentric phase

No main effect for the factor ‘contraction phase’ was found for the LD (p = 0.956) and GM (p = 0.089). No significant differences in mean LD and GM activity could be demonstrated between the concentric, isometric or eccentric phase of contraction, nor during the trunk, nor during the leg extension exercises (Table 2).

For all the paraspinal muscles no interactions between the main factors could be demonstrated, however a significant difference in mean EMG activity between the different contraction phases was noticed for all muscles separately.

Post hoc analysis for ‘contraction phase’ revealed that the LTT and LM activity was significantly higher during the concentric phase of the extension exercises compared to the eccentric contraction phase (respectively, p = 0.003 and p = 0.040). Whereas no significant differences in mean muscle activity between the concentric vs isometric phase and isometric vs eccentric contraction phase existed (p > 0.05).

Regarding the ILT, LTL and ILL significantly higher activity levels were found during the concentric contraction phase compared to the eccentric phase of contraction (p ≤ 0.001). Moreover a significant higher recruitment of these muscles during the isometric contraction compared to the eccentric phase of the dynamic-static extension exercises could be established (resp. p = 0.017; p = 0.002; p = 0.022).

Mean EMG levels (%MVC) for each contraction phase within the extension exercises are reported in Table 2.

### Borg score

The mean Borg score was significantly higher during trunk extension (15.5 ± 1.6) than during leg extension (13.8 ± 1.3) (p = 0.013). In addition there was a significant difference regarding the type of contraction. The rate of perceived exertion was higher during dynamic-static exercises (15.7 ± 1.6) than during dynamic exercises (13.7 ± 1.9) (p ≤ 0.001).

## Discussion

The present study was designed to investigate whether the amount of activity (%MVC) of the different parts of the posterior spine muscle chain is influenced by different extension exercise modalities. Therefore the mean muscle activity was analyzed during four different extension modalities.

The results of this study show that all muscles of the posterior chain were, given the intensity of 60% of 1 RM, active within the expected range during the different trunk and leg extension exercises in healthy individuals. The LD and GM however played a smaller role compared to the paraspinal muscles. The recruitment of the GM and LD during an extension movement of the spine can be clarified by the coupling between these muscles and the paraspinal muscles, which is formed through the fascia thoracolumbalis [50]. The lower activity levels of both GM and LD are in agreement with previous findings [21,26,28,32] and can be explained by the main function of these muscles, which is not back extension but arm and leg extension respectively. In contradiction with our results, other authors suggest a major role of the GM during trunk extension which is dependent upon the intensity of the exercise. These authors suggest that with increasing load and repetitions, the lumbar muscles become less responsible for maintaining the force output, while the GM becomes more powerful and responsible for the force output [40]. In the current study only 5 repetitions were investigated which was probably not sufficient enough to induce similar alterations in the muscle recruitment pattern.

These results indicate that for specific strengthening of the LD or the GM other exercises are more appropriate. Nevertheless, we showed that these muscles are contributing to the extension movement.

In literature, a wide variety of muscle activity levels during trunk and leg extension exercises are reported. Different exercise set –ups (starting angle, contraction modality, hand position) and used methods for measuring muscle activity (electrode placement) have been used, making comparisons between results difficult. In the current study mean thoracic and lumbar muscle activity ranged from 45 to 78% of the MVC. These findings are comparable with the findings for the studies of Arokoski et al. [21] and Ng et al. [26,33]. However, the observed activation of the lumbar spinal muscles is slightly higher than reported by Plamondon et al. [28]. The higher muscle activity in the present study could be explained by the difference in arm position between the studies. In the current study the arms were positioned further away from the center of gravity compared to the arm placement used in the study of Plamondon et al. [28], which resulted in a bigger lever arm and higher muscle recruitment [39].

Although the lumbar and thoracic paraspinal muscles can act synergistically to produce an extension force, several studies suggest that the back muscles are not one homogeneous muscle mass [32,51-53]. The back muscles are composed of different groups of fascicles with different functions. Therefore a distinction, based on anatomical and functional differences, between the thoracic and lumbar muscle groups is necessary. Both muscle groups cross the lumbar spine, whereas the lumbar muscle parts directly attach on to the lumbar vertebrae, the thoracic parts originate from the thorax and insert in long tendons that form the erector spinae aponeurosis [52]. The thoracic muscles, which are located more superficial, are be more force producing muscles, whereas the deeper lumbar muscles (especially the LM) tend to have a more specific stabilizing function of the spine. Therefore, we decided to investigate the thoracic (LTT and ILT) and lumbar extensor (LTL, ILL, LM) groups separately.

To our knowledge only few researchers have previously investigated the contribution of the LTT and ILT during extension exercises. The amount of thoracic muscle activity (45-64% MVC) in the current study is comparable with findings from previous reports during trunk extension in healthy people, although they did not make a distinction between the LTT or ILT as was done in the present study [18]. The necessity to make a distinction between these thoracic muscles has been demonstrated by Coorevits et al. [32], who showed that the LTT has a higher fatigue rate then the ILT during trunk extension in healthy people. Although the current study did differentiate between the thoracic muscles we did not find any differences between the thoracic muscles during performance of the extension exercise modalities which were previously described. The current study did reveal a higher contribution of the lumbar and thoracic muscles during trunk extension exercises than during leg extension exercise. To our opinion the difference can be attributed to the different kinematics and coupled muscle function between the two exercises. A trunk extension from departing from 45° trunk flexion can be seen as a dynamic pelvic and trunk movement. The leg muscles will extend the pelvis, the lumbar muscles will stabilize and extend the lumbar region on the pelvis, and the thoracic muscles will actually lift the trunk. On the contrary, with a fixed trunk in a horizontal position and the hips in a starting position of 45° flexion, most of the dynamic work is performed by the leg muscles while both back muscles groups deliver more static work. The back muscles need to stabilize the pelvis and spine to make leg lifting possible. Literature provides evidence that during concentric muscle work higher levels of activity are produced than during static work [30]. No earlier study has made the comparison in thoracic and lumbar muscle recruitment during both trunk and leg extension which emphasizes the relevance of the current study.

A homogeneous recruitment pattern of the lumbar muscles was observed during extension exercises. In agreement with Callaghan [18], we found the LM activity did no differ from ILL and LTL activity. However previous studies showed significant higher recruitment of the LM and the LTL, compared to the more lateral ILL, during trunk extension in healthy subjects [32,54]. In addition, using MRI, a previous study showed higher activity of the LM compared to ILL and LTL during trunk extension in chronic LBP patients [43]. Moreover Ng et al. found higher activity of the LM compared to Iliocostalis and Longissimus thoracis during respectively a trunk holding and leg holding test [26,33].

Possible explanations for the contradicting results are differences in exercise and measuring protocol. Coorevits et al. [32] objectified muscle fatigue whereas the present study measures the averaged muscle recruitment. Furthermore Coorevits et al. [32] and Ng et al. [26,33] studied muscle activity during isometric contraction, while in the present study dynamic and dynamic–static contractions were used. A second explanation of the homogeneous lumbar muscle usage found in the present study, could be the relative high intensity of the exercise (60% 1-RM). Since Mayer et al. [55] demonstrated that the contribution of the lumbar parts of the erector spinae compared to the LM was higher with increasing intensity, it is possible that in order to obtain a force output at 60% of the RM all the muscles are recruited at a comparable intensity. Therefore, further investigation regarding lumbar muscle activity in low load conditions is recommended. It is possible that, in agreement with the evidence of functional differences between the lumbar muscles [48,56], these low load conditions are more sensitive for differences in recruitment.

In contrast with a previous investigation [23], this study shows that lumbar muscle activity was higher during trunk than during leg extension. Discrepancies in exercises intensity and starting angle could explain the contradicting results. In the study of Plamondon et al. [23] the weight of the body part was not taken into account, which complicates the comparison with the current results. In the present study, based on the results of the pretest, all exercises were set at an equal intensity (60% of 1-RM) by adding weight or assisting the body part. Moreover, in the study of Plamondon et al. [28] leg extension was performed at 60° and trunk extension at 45° of flexion, while in our study both exercises were performed at 45° flexion. As suggested by Mannion et al. [57] changes in muscle length, induced by differences in starting angle, have a significant effect on force output of these muscles. Based on the Borg score, subjects experienced trunk extension as more intensive than leg extension, although the intensity of both exercises was equal. An explanation could be found in the muscles activity levels. Logically, because thoracic and lumbar muscles were recruited at a higher degree during trunk compared to leg extension, trunk extension was experienced as more fatiguing. The subjective feeling of heaviness, is normally determined by the weakest link. However, we did not inquire the region (upper, lower back or legs) of heaviness, so no judgment can be made about which muscle group is determining the feeling of heaviness. Further research into this aspect is warranted.

Our results also indicates that the modality of contraction (dynamic or dynamic-static) does not affect posterior muscle chain recruitment patterns. To our knowledge a comparison of back muscle activity between dynamic and dynamic-static extension exercises has not been investigated earlier. But in line with these results regarding muscle recruitment, Danneels et al. [22] found no difference in increase of the lumbar spinal muscle cross sectional area between dynamic and dynamic-static extension training.

Inspired by the basic principles of muscle training, when the goal of the exercises is to train muscles in terms of endurance, the intensity must be drawn up to a percentage of 60 [55]. The results of the current study show that when extension exercises are performed at 60% 1-RM, the amount of thoracic muscle activity during all exercises was comparable with the predetermined intensity. Therefore all types of extension exercises are suitable to improve the endurance capacity of the thoracic muscles. The level of lumbar muscle activity during leg extension exercises was also in agreement with this level of the exercise intensity (±60% 1-RM). On the contrary, during trunk extension, the amount of lumbar muscle activity clearly exceeded this level. This means that in clinical practice leg extension can be used to train lumbar muscle endurance, whereas trunk extension exercises at 60% of the 1-RM target the lumbar muscles at a higher training level.

The recruitment of the GM and LD remained far below 60%MVC, so to enhance the endurance of these muscles other exercises will be more appropriate.

Regarding the recruitment of the posterior muscle chain during the different phases of contraction, the present study showed higher levels of recruitment of all paraspinal muscles during the concentric compared to the eccentric contraction phase of the extension exercises. Higher muscle activation during concentric versus eccentric contraction was already demonstrated by other authors [23,58]. Plamondon et al. found the highest ES activity levels at L5/S1 near the horizontal position of the trunk, so during the concentric phase of the prone back extension exercises, and the lowest levels during the eccentric phase [23]. However, they did not report statistical significant differences. Moreover, Babault et al. reported lower activation levels of the knee-extensors during an eccentric compared to a concentric and isometric contraction of these muscles, which is probably due to a decreased voluntary activation during eccentric contractions [58]. Another explanation could be that during dynamic conditions there is a lower recruitment threshold, so full recruitment in dynamic conditions achieved at lower relative force levels compared to an isometric condition [59]. However, this statement cannot explain the higher activity levels of the ILT, LTL and ILL during isometric compared to eccentric contraction.

In the present study we studied a young healthy population. Since altered muscle activation patterns within specific populations are demonstrated [60], the results of the current study cannot be generalized to LBP patients.

## Conclusion

Our results demonstrated that recruitment of the posterior muscle chain during extension exercises at 60% 1-RM was influenced by the body part that was extended, but not by the type of contraction (dynamic or dynamic-static).

The activity of the thoracic extensors varied between 54% and 64%MVC during respectively leg and trunk extension, which is comparable with the premised intensity of 60% 1-RM. This suggests that to improve the endurance capacity of the LTT and ILT all four types of extension exercises could be used.

However, the activity of the lumbar muscle group exceeded the 60%MVC during the trunk extension exercises, whereas during leg extension the lumbar muscles were recruited less. This means that in clinical practice, therapists can use leg extension to ameliorate lumbar muscle endurance, whereas trunk extension exercises can be used to specifically activate the lumbar muscles and enhance their strength and endurance (70% 1-RM).

The LD en GM were activated at a low degree during all exercises, which implicates that to enhance the endurance capacity of these muscles other exercises than extension exercises, are more indicated.

## Competing interests

The authors declare that they have no competing interests.

## Authors’ contributions

EMDDR participated in the study design, in collecting the data, the statistical analyses, and drafting of the manuscript. AV, GGV and JOVO participated in the progress and drafting of the manuscript. LAD and JOVO participated in the interpretation of the statistical findings. LAD participated in the study design and in the progress and drafting of the manuscript. All authors read and approved the final manuscript.

## Pre-publication history

The pre-publication history for this paper can be accessed here:

http://www.biomedcentral.com/1471-2474/14/204/prepub
